# Knockdown of the fly spliceosome component *Rbp1* (orthologue of *SRSF1*) extends lifespan

**DOI:** 10.1007/s11357-025-02079-6

**Published:** 2026-01-09

**Authors:** Dan J. Hayman, Mirre J. P. Simons

**Affiliations:** School of Biosciences, University of Sheffield, Sheffield, UK

**Keywords:** *Rbp1*, * SRSF1*, Dietary restriction, mTOR suppression, Spliceosome

## Abstract

Biological regulation is an intricate process involving many layers of complexity, including at the RNA level. Alternative splicing is crucial in the regulation of which components of a protein-coding gene are spliced into a translatable mRNA. During ageing, splicing becomes dysregulated, and alternative splicing is implicated in disease and known anti-ageing treatments such as dietary restriction (DR) and mTOR suppression. In prior work, we have shown that DR and mTOR suppression modulate the expression of the spliceosome in the fly (*Drosophila melanogaster*). Here, we manipulated the five top genes that change in expression in both these treatments. We found that knockdown (using conditional in vivo RNAi in adults) of some spliceosome components rapidly induces mortality, whereas one, *Rbp1*, extends lifespan. Treatments that have more instant benefits on longevity are more translatable. We therefore subsequently repeated the *Rbp1* experiment but initiated *Rbp1* knockdown at later stages in adult life. We find that irrespective of the age of induction, knockdown of *Rbp1* extends lifespan. Our results posit the spliceosome itself as a hub of regulation that when targeted can extend lifespan, rendering it a promising target for geroscience.

**Supplementary Information:**

The online version contains supplementary material available at https://doi.org/10.1007/s11357-025-02079-6.

## Introduction

Ageing is characterised by a diverse range of molecular and cellular alterations and is the strongest risk factor for all age-related diseases [17, 32]. The geroscience hypothesis therefore states that if we can target and treat ageing, we will prevent all major debilitating age-related diseases [25]. The two best studied treatments which positively impact health and lifespan across species are dietary restriction (DR) and suppression of mammalian target of rapamycin (mTOR) [12, 16, 39, 49]. DR is conserved across evolution, spanning from yeast to mammals [10, 13]. It remains unclear, however, which precise nutrients enable the beneficial effects of DR and what the exact downstream mechanisms of DR are [14, 16, 47]. The beneficial lifespan-extending effects of suppression of mTOR appear to similarly be conserved across species [12], but downstream mechanisms again are not fully elucidated and are distributed across many different physiological and molecular pathways. For example, mTOR expression has been reported to both increase and decrease with age, depending on sex, tissue, and other specific conditions [3, 8, 39]. A shared feature of both mTOR suppression and DR is however that they both exert widespread effects on alternative splicing irrespective of species [20, 44, 48, 51]. Both mTOR suppression and DR may therefore orchestrate physiology that promotes healthy ageing through changes in genome-wide splicing.

Alternative splicing is the process regulating which transcribed components of a protein-coding gene are spliced into a translatable mRNA, determining a large proportion of the complexity of the proteome [34, 36]. Splicing changes rapidly in response to the environment [50] and patterns of splicing are heritable [28]. Alternative splicing is a major determinant of organismal complexity, and abnormal splicing events are implicated in disease and ageing [5, 34]. Indeed, a genome-wide dysregulation of alternative splicing is observed during ageing [22, 29, 30]. The molecular machinery through which splicing is carried out and regulated is known as the spliceosome, a large dynamic complex of approximately 100 different proteins, as well as snRNPs and small nuclear RNA (snRNA). Several other proteins trigger the assembly of the spliceosome, even if they are not themselves part of the spliceosome “core” [24, 60].

Each spliceosome component is recruited to the spliceosome as the complex forms around a 5′ splice site, and each component mediates specific parts of the splicing reaction [42, 62]. As each component is recruited (some individually, others as part of a complex), the spliceosome changes conformation and progresses through different stages of the splicing reaction [60]. Each component therefore has an important, albeit in some cases small, role to play in the splicing of mRNA. Previous studies have manipulated the expression of individual spliceosome genes and identified, importantly but also perhaps unsurprisingly, wide-reaching splicing and spliceosome regulatory changes [46]. Intriguingly, the manipulation of a single individual spliceosome component can modulate ageing; overexpression of one spliceosome component gene in *Caenorhabditis elegans*, *sfa-1*, extends lifespan, whereas knockdown of *sfa-1* negates the pro-longevity phenotypes of mTOR suppression and DR [20]. Modulating the spliceosome may thus have the potential to mimic the health benefits of both DR and mTOR suppression.

Here, we manipulated the spliceosome components that show the strongest transcriptomic changes in response to the rejuvenating effects of early-life mTOR suppression and DR in flies. Three spliceosome components truncated lifespan substantially when knocked down (*Sf3b1*, *barc* and *Prp5*), showing they are essential for life. One splicing factor, *Rbp1*, increased lifespan when knocked down and similarly increased lifespan when this knockdown was induced later in adult life. Modulation of the spliceosome therefore holds promise to achieve pro-longevity effects. As such, the spliceosome provides both a model to experimentally distil and a novel geroscience target to achieve pro-longevity effects on a whole organism level.

## Methods

### Drosophila conditions

Fly media was composed of the following components, as previously described [19]: 6% cornmeal, 13% table sugar, 1% agar, 0.225% nipagin and 8% yeast (all w/v), with the addition of 0.4% (w/v) propanoic acid (Sigma-Aldrich) for fly growing bottles only. All experiments utilised the near globally expressed *daughterless*-*GeneSwitch* (daGS) driver [56] to conditionally drive transgenes in vivo, thereby removing the potential effect of background genotype [19]. In experimental conditions requiring activation of daGS, RU486 (200 µM; Thermo Fisher Scientific) was supplemented and dissolved in ethanol, and control treatments received the same amount of ethanol. Media was split from the same batch to ensure the exact same food was used for both treatments. Previous experiments in our laboratory have found no effect of RU supplementation on lifespan using this same driver line [9, 13, 19]. All flies were maintained and grown at 25 °C. The fly lines used are shown in Table S3.

### Survival experiments

Flies were kept for mating for 2 days after eclosion, before being sorted under light carbon dioxide anaesthesia, with females put into cages to assess survival as we described previously [13, 19, 35, 41]. Cox proportional hazard mixed (“coxme”) effects models were used to analyse longevity data and included cage as a random effect and date of birth as a fixed effect to correct for shared environment effects. Coxme interval-based models were used to assess whether activation of *Rbp1* RNAi affected lifespan in later life. These models allow time-dependent covariates and estimate differential risk between control-treated and *Rbp1* RNAi activation at different ages. We used 12 days as the period required to activate the GeneSwitch and RNAi construct fully, as we found this is the timepoint when transgene activation was maximised [48]. Activation was coded as a three-level factor (control, early and late). These statistical models are designed to test for age-corrected current treatment effects compared to control [35, 54].

### RNA isolation and qPCR

In order to validate the knockdown of *Rbp1*, which extended lifespan in two independent experiments (Figs. 2 and 3), and *Prp5*, which reduced lifespan, RNA was extracted from flies fed RU-supplemented or control food (RNeasy Mini Kit, Qiagen; *n* ≥ 4 samples per condition, with 3 whole flies per sample). A total of 300 ng of RNA from each sample was reverse transcribed using the PrimeScript FAST RT reagent kit with gDNA eraser (Takara). The TB Green Premix Ex Taq II FAST qPCR kit (Takara, using the manufacturer’s recommended qPCR cycle protocol) was used to assess gene expression of *Rbp1* and *Prp5* relative to the gene encoding β-actin (*Act5C*) by quantitative real-time PCR, using the 2^−ΔCT^ method to normalise results (Figure S2). The following primers were used (all shown 5′ to 3′): *Rbp1* forward (TCCGGACGCTACAGGATAACTC), *Rbp1* reverse (TTGAAGGTTGCTGGCTGTGG), *Prp5* forward (GTGGAAAACGCGACGATAAGC), *Prp5* reverse (TCCTTCTCGCGCTCCTTTTC), *Act5C* forward (ACACAAAGCCGCTCCATCAG) and *Act5C* reverse (TGTCGACAACCAGAGCAGCA). For both genes, we found similar magnitudes of reduction, highlighting the effectiveness of this conditional RNAi approach.

### Data mining from two transcriptomic experiments

#### RNA isolation and sequencing

Samples of ~4 whole female flies were collected and snap-frozen. RNA was extracted from a lysate generated using bead milling using RNeasy mini kits (Qiagen) as per the manufacturer’s instructions. RNA quality and concentration was assessed prior to sequencing using a TapeStation (Agilent). Samples were then shipped on dry ice to the Oxford Genomics Centre where samples were reverse-transcribed and an equal concentration was polyA-enriched library prepped and deep-sequenced in full multiplex using Illumina HiSeq4000 with 75 bp paired ends.

#### Description of the two experiments

The transcriptome data presented was extracted from two prior experiments with more detailed publications forthcoming. These experiments used mated females and an identical protocol for all fly husbandry as reported in the current manuscript. Furthermore, these experiments are described in a preprint [48] where we first found a connection between mTOR signalling and the spliceosome. The DR transcriptomes are part of work described in a PhD thesis [7]. These two studies are currently in preparation for submission. For purposes of this present study, we mined the data related to the spliceosome.

The mTOR data is based on experiments where we knocked down mTOR in early adulthood only and found that this had a sustained longevity benefit. These used RNAi for mTOR from the TRiP collection [37] and the same GeneSwitch driver. The transcriptomic signature analysed here is from an experiment involving 32 samples across two timepoints (early and late adulthood, age 27 and 39 days, respectively) in four treatments (transient early and late knockdown, continuous knockdown and control). The transcripts reported here are from a glmQLFit model using edgeR extracting the transcriptional change uniquely associated with early life mTOR knockdown, measured at old age. We previously found that this pro-longevity treatment led to an enrichment of the spliceosome [48].

The DR data is based on transcriptomes from ywR flies [41] that were subjected to a DR switching paradigm [7, 35, 59]. We extracted transcripts that responded to the switch to DR from fully fed conditions (8% vs 2% yeast diets) [35], as measured 48 h after the dietary change. The fly uniquely responds to DR by lowering its mortality risk almost instantly [15, 33, 35]. The transcriptional change observed during this period could therefore be uniquely associated with the anti-ageing effects of DR. Flies were switched to DR at age 19–20 days and sampled 48 h thereafter. This experiment consisted of 10 samples in each diet category.

#### Bioinformatics

Reads were mapped to the *Drosophila melanogaster* genome (Release 6) using HISAT2 (version 2.2.1) [40]; transcripts were assigned and counted using stringtie (version 1.3.4) [40]. We used the annotated (and thus not de novo assembled) transcriptome of the fly. Data were loaded into R using ballgown [40] and analysed for differential expression using edgeR [45] using the general linear modelling framework in glmQLFit. For Gene Ontology (GO) enrichment analyses, we used TopGO [2], using the “weight01” method to test for statistical significance, which allows for dissection of related terms by taking into account the GO hierarchy structure. Pearson’s Chi-squared test with Yates’ continuity correction was used to test in each experiment for enrichment of spliceosome genes relative to the expected number based on the number of genes annotated to the spliceosome-related GO terms.

## Results

### The expression of spliceosome genes change consistently between DR and transient mTOR suppression

**Fig. 1:**
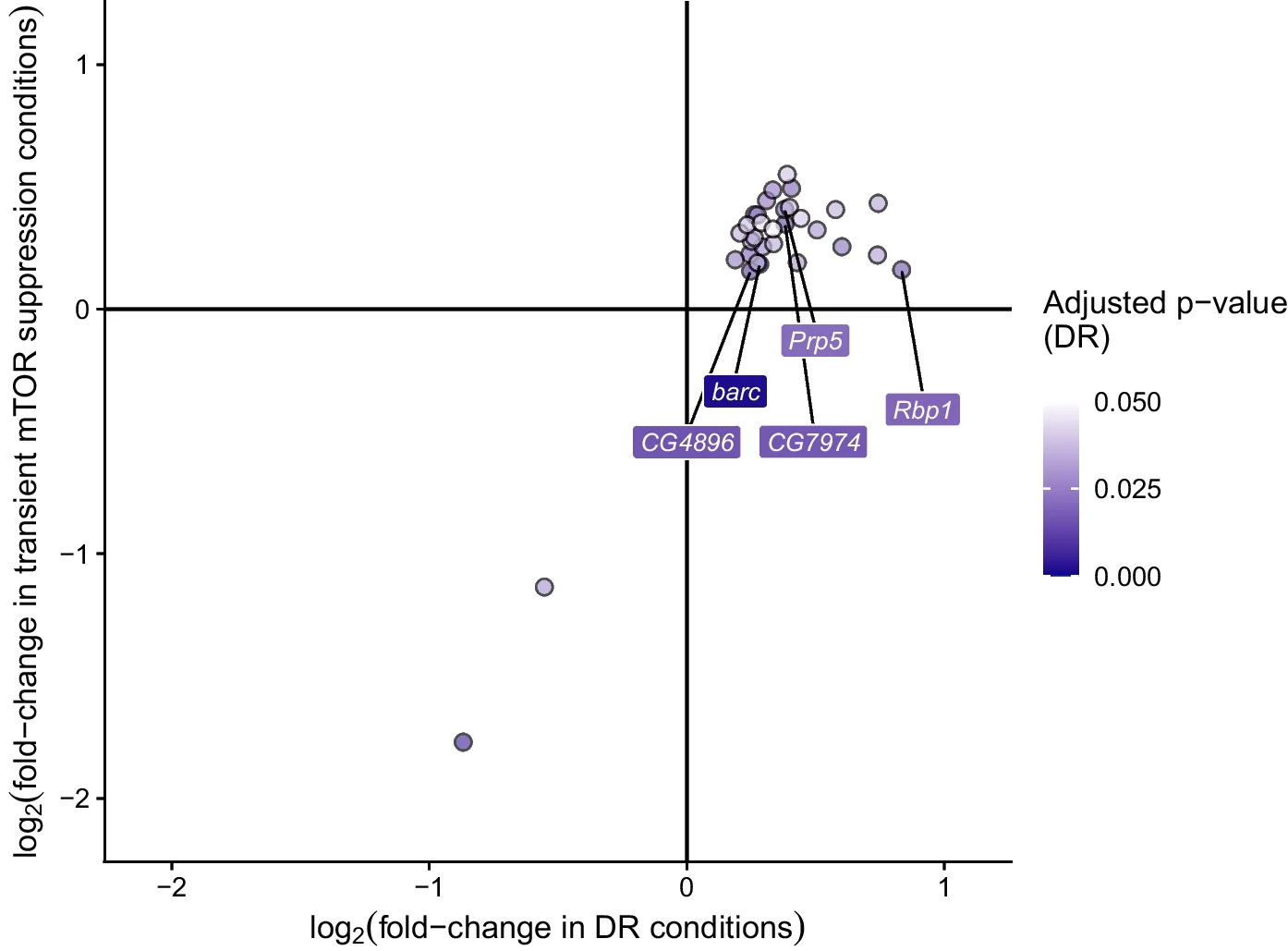
The spliceosome is modulated in both DR and transient mTOR suppressed conditions. Spliceosome genes which respond similarly between DR and transient mTOR suppression are plotted. In total 32 spliceosome-annotated genes were significantly differentially expressed (adjusted *p*-values ≤ 0.05) and showed concordant transcriptional change (in terms of log_2_ fold-change) in response to transient mTOR suppression and DR in *Drosophila melanogaster*. The vast majority of these (30 of the 32) were upregulated. The five genes from this set we subsequently tested for lifespan phenotypes are labelled and represent the top 5 most significantly upregulated genes in the DR treatment that were also significantly upregulated in response to transient mTOR suppression. We used the DR *p*-values as the discriminator here as in comparison to the mTOR dataset there were fewer spliceosome genes differentially expressed. The fill colour here represents the adjusted *p*-value of each gene in the DR treatment

We tested using previously generated transcriptomes in our group whether the spliceosome changed concordantly between both DR and transient suppression of mTOR. Suppression of mTOR in early adult life (using RNAi) has long-lasting benefits, which we have previously suggested are mediated via the spliceosome [48]. We combined this dataset with a transcriptome measuring the response to DR within 48 h. Considering that the fly responds rapidly to DR in terms of a reduction in mortality rate [15, 33, 35, 59], the changes occurring in our 48 h response dataset during this time should be devoid of the longer-term compensatory effects which are not causal of the observed lifespan extension. Where previous studies of this paradigm used microarrays [59], we now used next-generation sequencing. Gene Ontology (GO) enrichment analysis revealed that spliceosome-related GO terms (identified as genes which were in Gene Ontology terms featuring the word “spliceosome”, see Table S1) were highly significantly enriched in both experiments (Figure S1). Furthermore, there was a significant enrichment of differentially expressed spliceosome-related genes (adjusted *p*-value < 0.05) in each experiment relative to the expected number based on how many genes were annotated to spliceosome-related GO terms (DR: *χ*^2^ = 151.2, df = 1, *p*-value < 2.2 × 10^–16^; transient mTOR suppression: *χ*^2^ = 3287.9, df = 1, *p*-value < 2.2 × 10^–16^). We compared whether the same spliceosome or spliceosome-regulatory genes were differentially expressed in both the mTOR and DR datasets and whether they were modulated with concordant directionality. Of the 280 total genes annotated to spliceosome-related Gene Ontology terms, 187 genes were significantly differentially expressed upon transient mTOR suppression treatment and 35 upon DR. Thirty-two of these genes were differentially expressed with the same directionality in both DR and transient mTOR suppressed conditions (Fig. 1 and Table S2).

### Individual spliceosome components modulate lifespan

**Fig. 2:**
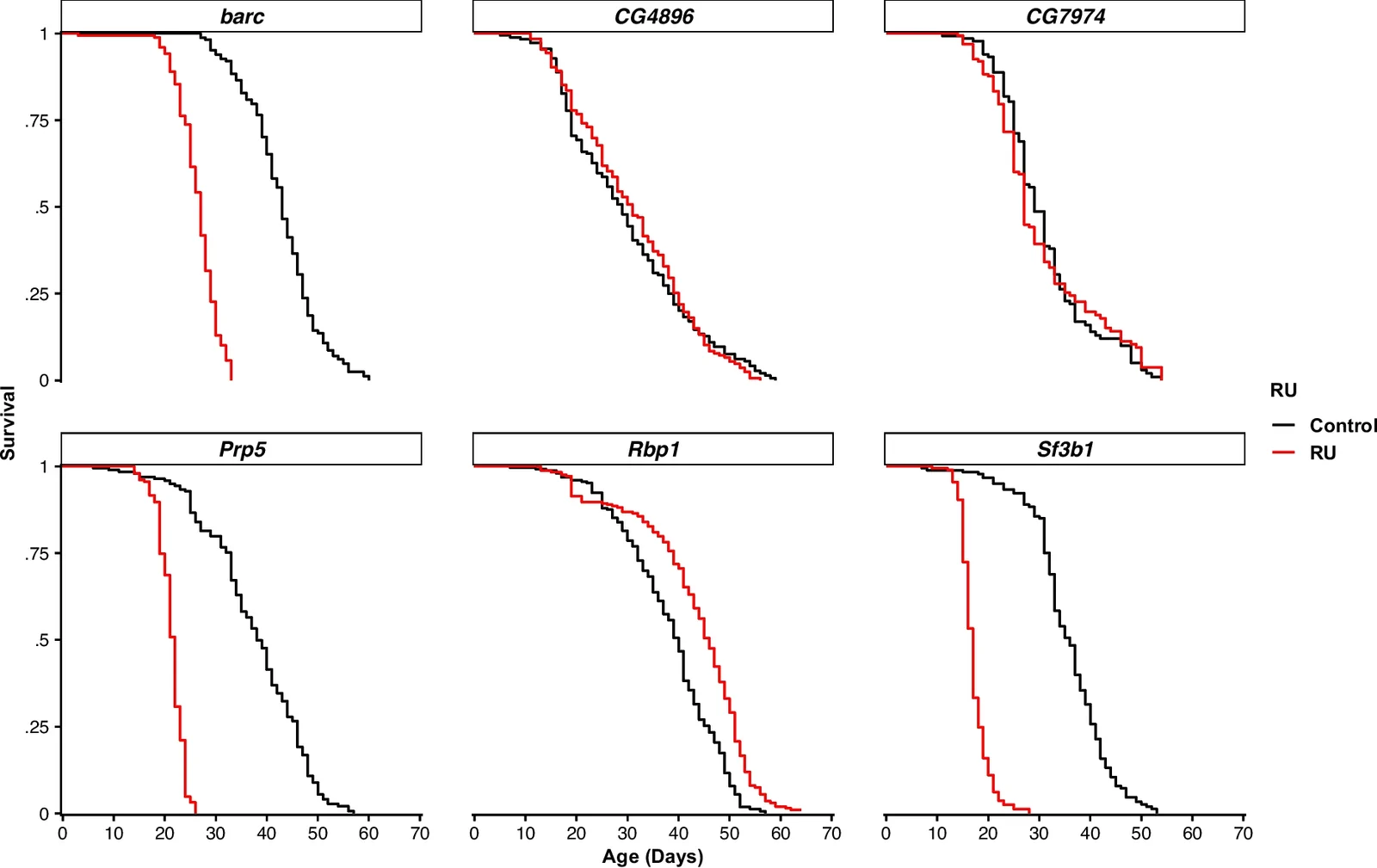
Knockdown of spliceosome genes modulates lifespan. Driving spliceosome gene RNAi with the *daughterless*-*GeneSwitch* (daGS) global conditional driver in female flies from the age of 4 days post-eclosion onwards reveals that different spliceosome components modulate lifespan in different directions (*n* ≥ 133 flies per condition, total *N* per genotype ≥ 296). GeneSwitch activity is initiated by supplementation with RU486 (RU). Hazard ratios and *p*-values for the experiment are shown in Table 1

To test whether the spliceosome components associated with both pro-longevity treatments were able to modulate lifespan, we knocked them down in adults using in vivo RNAi on rich diets [13]. There is a close to complete genome-spanning in vivo RNAi library that we used for this purpose [37]. The pro-longevity-associated spliceosome genes were upregulated in response to DR and so the classic interpretation would be they may be required to sustain life or the DR-longevity benefit. In such a scenario, no effect or a reduction of lifespan is expected under fully-fed conditions. However, if the spliceosome is upregulated as a compensatory response to DR, an extension of lifespan can be expected on rich diets [13]. Therefore, the prediction of how the expression of these genes associates to lifespan and on which diet is equivocal. We therefore started our screen on rich diets, on which flies live relatively short, and used RNAi for convenience, as these are readily available from stock centres. We tested the top 5 most significantly upregulated genes within the DR dataset that were also upregulated and significantly differentially expressed in the transient mTOR suppression dataset. We chose to select using the significance from the DR experiment, as in comparison to the transient mTOR suppression treatment, fewer spliceosome components changed in expression (35 compared to 187). The DR dataset thus overall appeared less sensitive, or DR had a less widespread impact on the spliceosome. We additionally filtered for only spliceosome components which increased in expression in response to both pro-longevity treatments, as this was the general direction of transcriptional change. We further included *Sf3b1* as it is a known important spliceosome component and has previously been investigated in relation to cancer proliferation and mTOR signalling [11, 18]. Although knockdown of *CG4896* and *CG7974* did not affect survival, knockdown of *barc*, *Prp5* and *Sf3b1* each significantly reduced lifespan, whilst *Rbp1* knockdown significantly extended lifespan (Fig. 2 and Table 1).

**Table 1:** Statistics for knockdown of spliceosome genes. Data was analysed using Cox proportional hazard mixed effects models using right-censoring where applicable [54, 55], incorporating cage as a random effect and experimental batch as fixed effect to correct for shared environmental effects from housing and growing conditions. Negative log hazard ratios indicate an increase in lifespan

Gene RNAi	log_e_ (hazard ratio)	Standard error	*N*	*p*-value
*barc*	3.90	0.33	340	< 0.0001
*CG4896*	−0.14	0.11	375	0.20
*CG7974*	0.24	0.23	296	0.30
*Prp5*	3.44	0.24	404	< 0.0001
*Rbp1*	−0.69	0.13	501	< 0.0001
*Sf3b1*	4.35	0.33	388	< 0.0001

### Knockdown of Rbp1 reduces mortality when initiated later in adult life

**Fig. 3:**
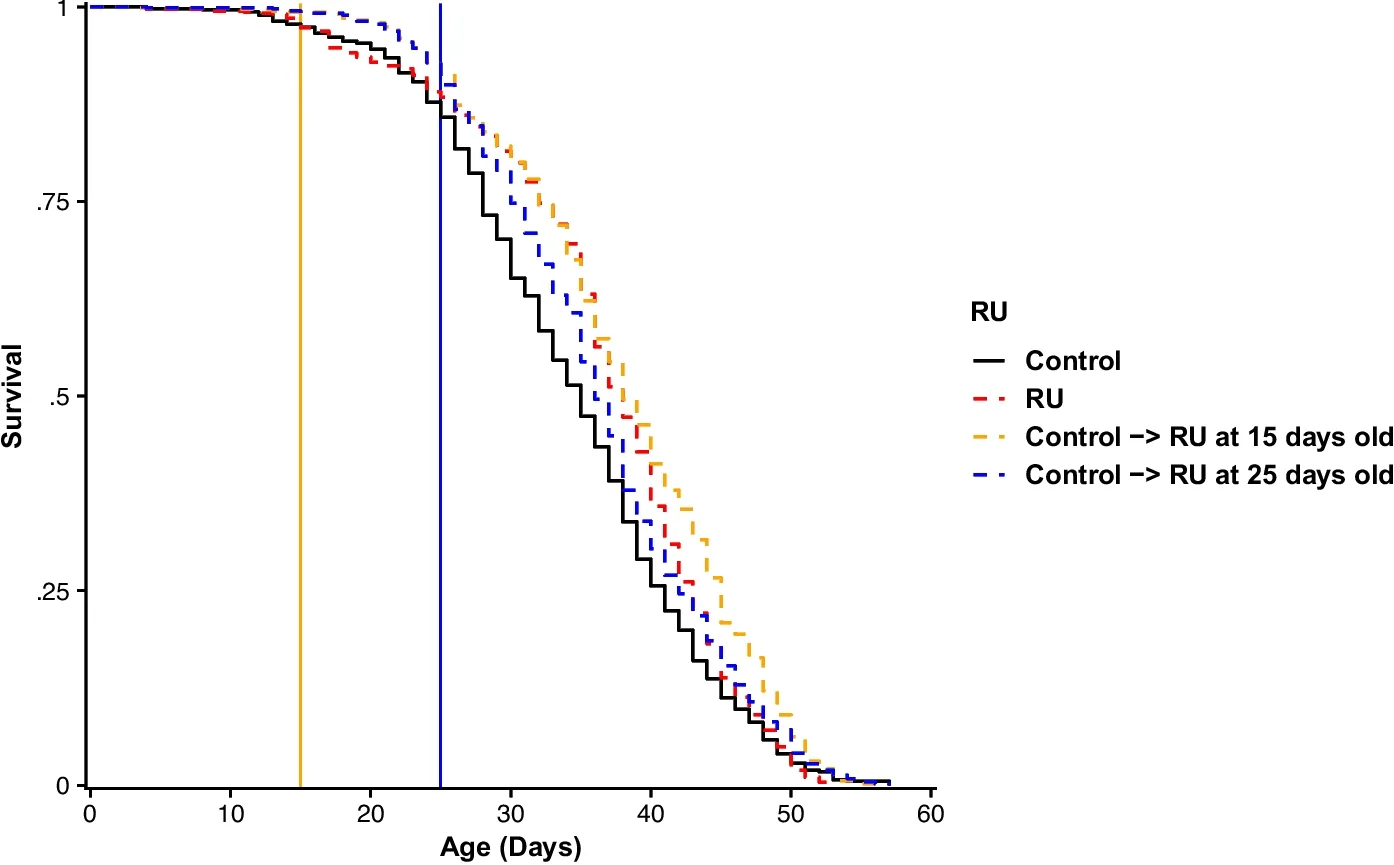
Knockdown of *Rbp1* reduces mortality risk irrespective of when it is triggered. Conditional activation of *Rbp1* RNAi reduced mortality irrespective of whether it was induced 4 days post-eclosion (as in Fig. 2) or at later timepoints; 15 days post-eclosion or 25 days post-eclosion (*n* ≥ 700 flies per condition, total *N* per comparison ≥ 1525, coxme interval-based model). The model used to calculate statistics treats all flies as controls until 12 days after the point of switching to RU (to maximise transgene activation [48]), at which point flies become part of their respective treatment groups. This ensures that any early mortality which happens before the RNAi is activated will not affect the statistical outcome. The solid vertical yellow and blue lines signify the timepoints of RU supplementation

Considering that conditionally driving *Rbp1* RNAi from early adult life extended lifespan, we wanted to test whether this treatment could also extend lifespan when activated in later life, as is found with, for example, DR [58]. We have previously found that triggering RNAi using *daughterless*-*GeneSwitch* is able to efficiently suppress the target gene regardless of the age at which it is induced [48]. We used this same experimental paradigm to repeat the *Rbp1* knockdown experiment with two additional conditions: *Rbp1* knockdown from 15 days onwards and *Rbp1* knockdown from 25 days onwards. Both of these treatments reduced mortality risk (Fig. 3 and Table 2). We confirmed the effectiveness of *Rbp1* knockdown using RT-qPCR (Figure S2). RU supplementation has no effect on lifespan in our hands [9, 13, 19]. To confirm this again, we crossed the *daughterless*-*GeneSwitch* driver line to a line in which an empty RNAi vector was integrated at the same insertion site as the *Rbp1* RNAi line and found no effect of RU supplementation on longevity (Figure S3). Knockdown of *Rbp1* can therefore improve life expectancy of flies irrespective of when the knockdown occurs.

**Table 2:** Statistics for *Rbp1* knockdown at different timepoints or overall. Data was analysed using coxme interval-based models, incorporating the cage ID as a random effect and experimental batch to capture shared environmental effects. As treatment was initiated in later adult life, we coded this as a time-dependent covariate in the coxme models [35, 53]

Comparison	log_e_ (hazard ratio)	Standard error	*N*	*p*-value
Overall RU vs control	−0.23	0.06	2800	0.00012
RU at 15 days vs control	−0.30	0.08	1525	0.00017
RU at 25 days vs control	−0.23	0.09	1547	0.00590

## Discussion

Half of the tested individual spliceosome components negatively affected lifespan when experimentally reduced, but interestingly, *Rbp1* increased lifespan when knocked down. The direction of this effect is surprising as both pro-longevity treatments increase *Rbp1* expression; *Rbp1* knockdown alone is able to increase lifespan, whilst in the context of pro-longevity treatments, its increased expression is correlated with increased lifespan. This contradiction could be explained by interactions within the spliceosome or, alternatively, the spliceosome response observed is a compensatory response and it is actually the reduction of its downstream effects, possibly a reduction in overall translation to protein, that extends lifespan. We recently observed similar compensatory effects for a range of DR-responsive genes, where similarly genes that increased in expression, when knocked down, increased lifespan [13].

In vertebrates, the inhibition of many spliceosome genes severely affects many essential cellular processes, and mutations in spliceosome genes are frequently observed in cancers [38, 52]. To a certain extent, it was therefore unsurprising that we observed increased mortality upon conditional knockdown of *barc*, *Prp5* and *Sf3b1* in flies. However, the lifespan extension upon conditional knockdown of the SR protein-encoding gene *Rbp1* was unexpected. In theory, knockdown of *Rbp1* should reduce spliceosome assembly at *Rbp1* binding sites in specific exonic splicing enhancers, which therefore should negatively impact splicing fidelity of a subset of mRNAs. The mechanistic reason for this positive effect of knockdown of *Rbp1* on lifespan is at present unclear.

The spliceosome components which rapidly increased mortality risk when knocked down in this study are all recruited to the spliceosome at a later stage than the point at which Rbp1 plays a role; barc, Sf3b1 and Prp5 are all recruited at the pre-spliceosome stage (also known as Spliceosomal Complex A). Prp5 is recruited in order to bridge the U1 and U2 snRNPs together at the pre-mRNA [31, 61], whilst barc and Sf3b1 are recruited as part of the U2 snRNP [1, 57]*.* In contrast, *Rbp1* encodes an SR protein, a class of proteins which bind pre-mRNA and recruit early spliceosome components to splice sites even before the commitment complex (Spliceosomal Complex E) has formed [23, 26). Therefore, perhaps a reduction (but not complete ablation) of spliceosome assembly, such as that which results from *Rbp1* suppression, benefits organismal health and longevity. One potential mechanism could be improved proteostasis by causing overall reduced protein synthesis [4, 21]. Perhaps suppression of later-stage spliceosome components causes the spliceosome to stall fully on the mRNA, leading to a complete failure to effectively translate protein and thus resulting in a severely truncated lifespan.

An important result from this study is that *Rbp1* knockdown extends lifespan also when instigated later in adult life. Geroscience-based treatments which do not require lifelong treatment will be far easier to translate to the clinic [27]. So far, only mTOR suppression may be capable of achieving this across organisms [6, 12, 48]. *Rbp1* is especially interesting in this aspect as its human orthologue (*SRSF1*) [26] has also previously been identified independently using a cell-based screen on reprogramming and produces beneficial phenotypes in other organisms, including mice [43]. More generally, individual spliceosome components, rather than the spliceosome as a whole, may prove powerful targets for healthspan-modifying drugs.

## Supplementary Information

Below is the link to the electronic supplementary material.

ESM 1 (PDF 5.72 KB)

ESM 2 (PDF 6.51 KB)

ESM 3 (PDF 5.19 KB)

ESM 4 (DOCX 3.34 MB)

ESM 5 (XLSX 36.3 KB)
