# Supplementary material for: Knockdown of the fly spliceosome component Rbp1 (orthologue of SRSF1) extends lifespan
Source: GeroScience. 2026 Jan 9;48(6):7805–14. doi: 10.1007/s11357-025-02079-6 (PMC13638910; doi:10.1007/s11357-025-02079-6)
Supplement: Supplementary file 1 [file 11357_2025_2079_MOESM1_ESM.pdf]

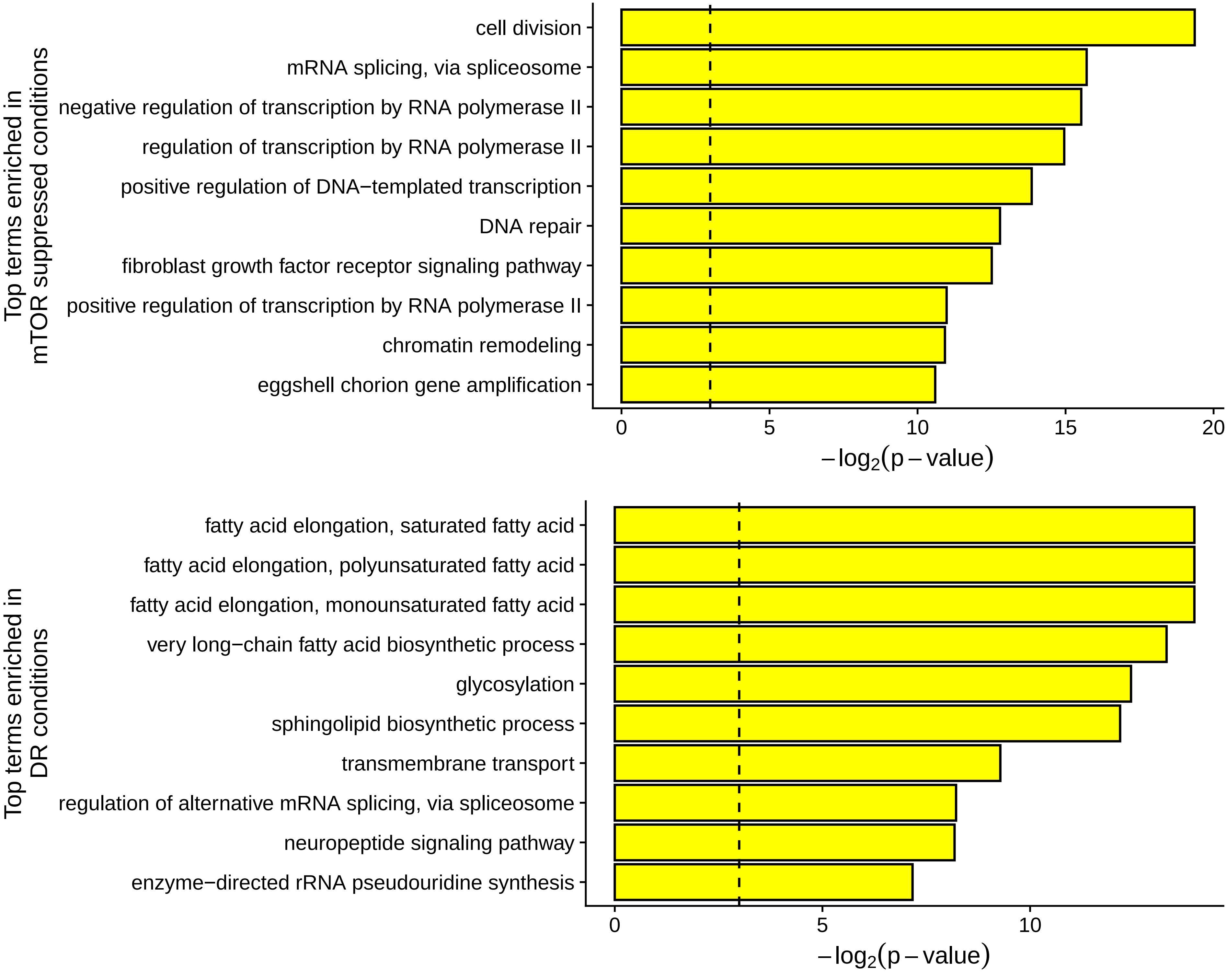

Top terms enriched in mTOR suppressed conditions
cell division
mRNA splicing, via spliceosome
negative regulation of transcription by RNA polymerase II
regulation of transcription by RNA polymerase II
positive regulation of DNA−templated transcription
DNA repair
fibroblast growth factor receptor signaling pathway
positive regulation of transcription by RNA polymerase II
chromatin remodeling
eggshell chorion gene amplification
0
5
10
15
20
−log2(p − value)
Top terms enriched in DR conditions
fatty acid elongation, saturated fatty acid
fatty acid elongation, polyunsaturated fatty acid
fatty acid elongation, monounsaturated fatty acid
very long−chain fatty acid biosynthetic process
glycosylation
sphingolipid biosynthetic process
transmembrane transport
regulation of alternative mRNA splicing, via spliceosome
neuropeptide signaling pathway
enzyme−directed rRNA pseudouridine synthesis
0
5
10
−log2(p − value)
