# Supplementary material for: Knockdown of the fly spliceosome component Rbp1 (orthologue of SRSF1) extends lifespan
Source: GeroScience. 2026 Jan 9;48(6):7805–14. doi: 10.1007/s11357-025-02079-6 (PMC13638910; doi:10.1007/s11357-025-02079-6)
Supplement: Supplementary file 2 [file 11357_2025_2079_MOESM2_ESM.pdf]

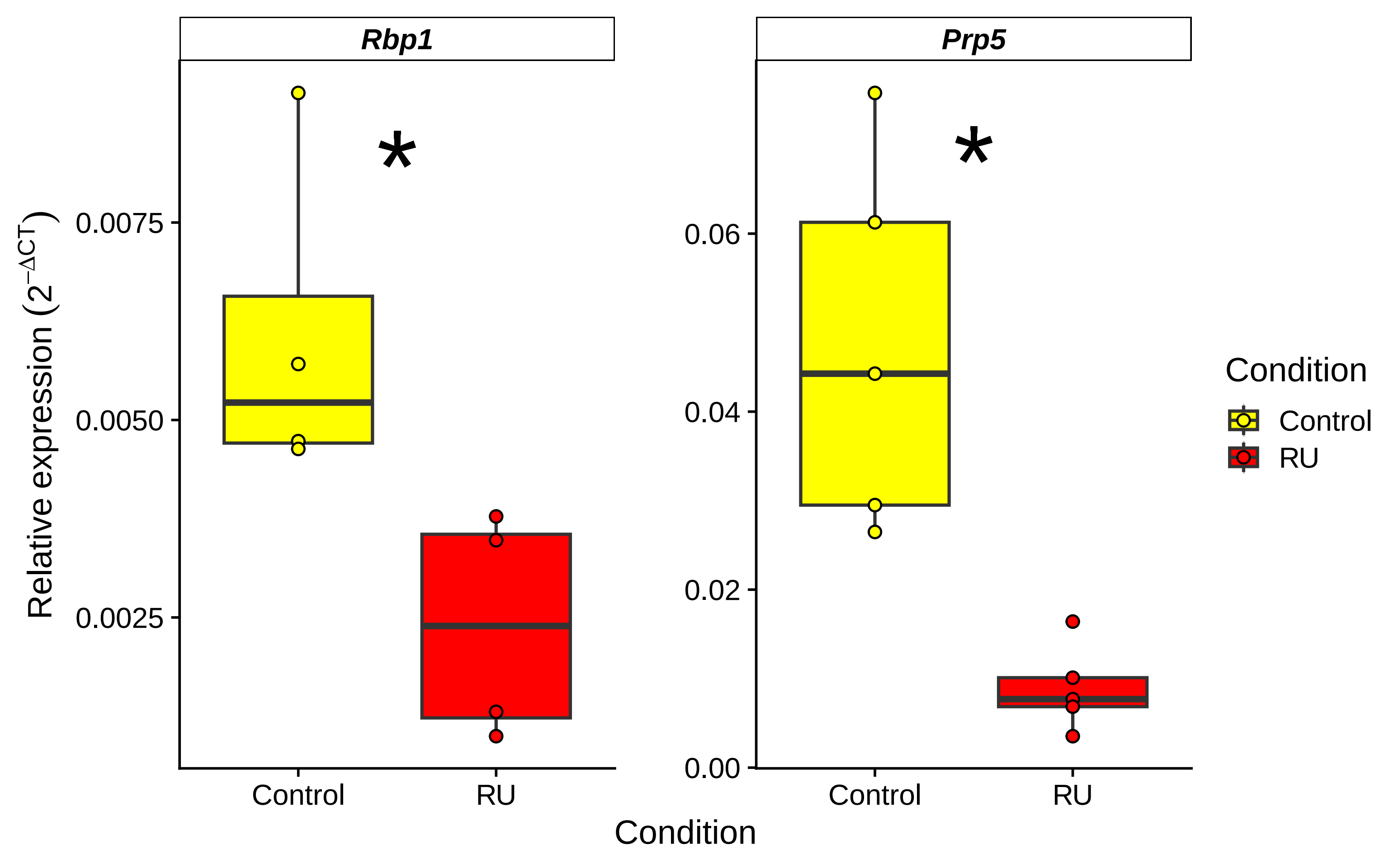

Rbp1
Prp5
*
*
Relative expression ($2^{-\Delta CT}$)
0.0075
0.0050
0.0025
0.06
0.04
0.02
0.00
Control
RU
Control
RU
Condition
Condition
Control
RU
