# Supplementary material for: Knockdown of the fly spliceosome component Rbp1 (orthologue of SRSF1) extends lifespan
Source: GeroScience. 2026 Jan 9;48(6):7805–14. doi: 10.1007/s11357-025-02079-6 (PMC13638910; doi:10.1007/s11357-025-02079-6)
Supplement: Supplementary file 3 [file 11357_2025_2079_MOESM3_ESM.pdf]

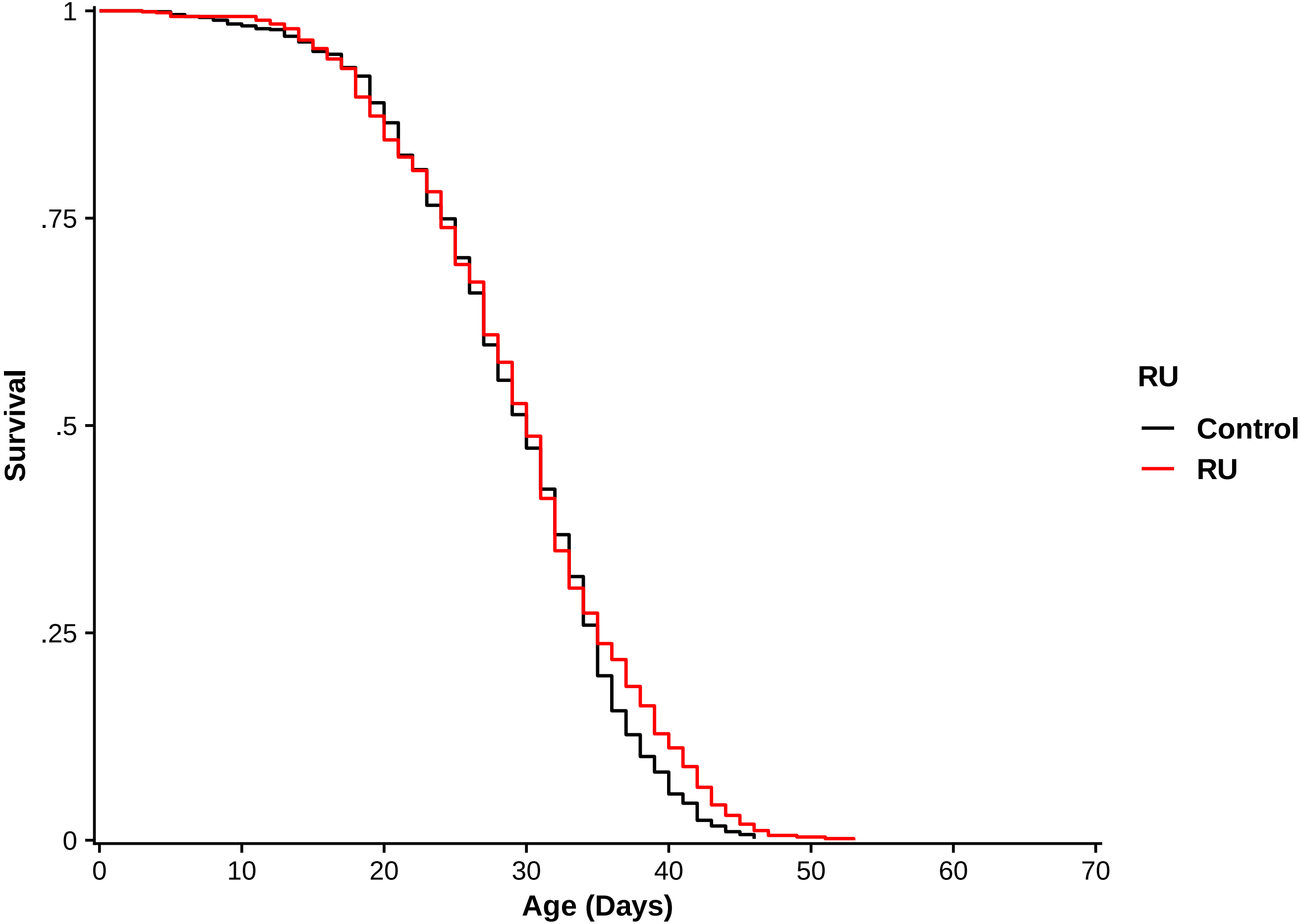
1
.75
.5
.25
0
Survival
0
10
20
30
40
50
60
70
Age (Days)
RU
Control
RU
