# Supplementary material for: Knockdown of the fly spliceosome component Rbp1 (orthologue of SRSF1) extends lifespan
Source: GeroScience. 2026 Jan 9;48(6):7805–14. doi: 10.1007/s11357-025-02079-6 (PMC13638910; doi:10.1007/s11357-025-02079-6)
Supplement: Supplementary file 4 [file 11357_2025_2079_MOESM4_ESM.docx]

Supplementary material

**Table S1 -** Spliceosome-related Gene Ontology terms used to filter shared genes changing in mTOR suppressed and DR conditions.

Generation of catalytic spliceosome for first transesterification step

Generation of catalytic spliceosome for second transesterification step

Trans assembly of SL-containing precatalytic spliceosome

Cis assembly of pre-catalytic spliceosome

mRNA trans splicing, via spliceosome

Alternative mRNA splicing, via spliceosome

Regulation of alternative mRNA splicing, via spliceosome

Spliceosome conformational change to release U4 (or U4atac) and U1 (or U11)

mRNA splicing, via spliceosome

mRNA cis splicing, via spliceosome

Regulation of mRNA splicing, via spliceosome

Negative regulation of mRNA splicing, via spliceosome

Positive regulation of mRNA splicing, via spliceosome

U2-type prespliceosome

U2-type precatalytic spliceosome

U2-type catalytic step 1 spliceosome

U2-type catalytic step 2 spliceosome

Prespliceosome

Precatalytic spliceosome

Catalytic step 1 spliceosome

Catalytic step 2 spliceosome

U12-type prespliceosome

U12-type precatalytic spliceosome

U12-type catalytic step 1 spliceosome

U12-type catalytic step 2 spliceosome

Spliceosome-depend formation of circular RNA

U2-type prespliceosome assembly

Regulation of mRNA cis splicing, via spliceosome

Negative regulation of mRNA cis splicing, via spliceosome

Positive regulation of mRNA cis splicing, via spliceosome

**Table S2 - The list of 280 genes annotated to the GO terms shown in Table S1.** Genes are ordered by adjusted p-value from the DR experiment, but log_2_ fold-changes and adjusted p-values for both the DR and transient mTOR suppression experiments are shown. Also shown are the spliceosome-related GO terms each gene is annotated to. 30 spliceosome-annotated genes are upregulated upon both transient mTOR suppression and DR, whilst 2 spliceosome-annotated genes are downregulated in both treatments. The top 5 most significantly upregulated genes from this list by adjusted p-value in the DR experiment, but which were also significant in the transient mTOR suppression experiment, were selected to knock down *in vivo* using RNAi. These genes are shown in bold.

(TABLE S2 IS UPLOADED AS A SEPARATE FILE)

**Table S3 - Fly lines used in this manuscript, with source stock and/or publication.**

| **Line** | **Full genotype** | **Source or Bloomington *Drosophila* Stock Centre number** | **Reference** |
| --- | --- | --- | --- |
| *daughterless*-GeneSwitch (daGS) | y[1] w[*]; daGS | Gift from Marc Tatar | (Tricoire et al., 2009) |
| UAS-*barc* RNAi | y[1] v[1]; P{y[+t7.7] v[+t1.8]=TRiP.HMJ02069}attP40 | 42504 | (Ni et al., 2011) |
| UAS-*CG4896* RNAi | y[1] v[1]; P{y[+t7.7] v[+t1.8]=TRiP.HMJ24125}attP40 | 62885 |  |
| UAS-*CG7974* RNAi | y[1] v[1]; P{y[+t7.7] v[+t1.8]=TRiP.GLC01718}attP2 | 50596 |  |
| UAS-*Rbp1* RNAi | y[1] sc[*] v[1] sev[21]; P{y[+t7.7] v[+t1.8]=TRiP.HMC03902}attP40 | 55688 |  |
| UAS-*Prp5* RNAi | y[1] v[1]; P{y[+t7.7] v[+t1.8]=TRiP.HMJ24050}attP40 | 62507 |  |
| UAS-*Sf3b1* RNAi | y[1] v[1]; P{y[+t7.7] v[+t1.8]=TRiP.HM05176}attP2 | 28965 |  |
| TRiP control line (attP40) | y[1] v[1]; P{y[+t7.7]=CaryP}Msp300[attP40] | 36304 |  |
| UAS-*mTOR* RNAi | y[1] sc[*] v[1]; P{y[+t7.7] v[+t1.8]=TRiP.HMS01114}attP2 | 34639 |  |

**Figure S1 - Spliceosome-related GO terms are enriched in both the mTOR suppression and DR experiments.** TopGO was used to enrich the mTOR suppression and DR RNA-sequencing experiments for GO terms using the weight01 method to test for statistical significance, which allows for dissection of related terms by taking into account GO hierarchy structure. The top 10 most significantly enriched GO terms for each experiment are shown. “mRNA splicing, via spliceosome” was the second most significantly enriched term in the mTOR suppression experiment, and both “regulation of alternative splicing, via spliceosome” and “spliceosomal snRNP assembly” were also significantly enriched, albeit not in the top 10 enriched GO terms. “Regulation of alternative mRNA splicing, via spliceosome” was in the top 10 most enriched terms for the DR experiment, and “spliceosomal complex disassembly” was also significantly enriched, but not in the top 10 most highly enriched terms.

**Figure S2 - Validation of *Rbp1* and *Prp5* knockdown upon RU supplementation.** At least 4 samples from each condition (3 flies per sample) were collected 12 days after flies were first provided with control or RU-supplemented food. RNA was extracted, reverse transcribed and subsequently *Rbp1* and *Prp5* levels were measured relative to *Act5C* (encodes β-actin) by quantitative real-time PCR, using the 2^-ΔCT^ method to normalise expression. Mean relative expression of *Rbp1* was reduced by approximately 2.5-fold in flies fed RU-supplemented food relative to controls (p = 0.033, n = 4 samples per condition), whilst *Prp5* expression was reduced by approximately 5.3-fold (p = 0.013, n = 5 samples per condition).

**Figure S3 - There is no effect of RU486 supplementation on lifespan in flies where the *da*-*GeneSwitch* driver is crossed to a line in which an empty RNAi vector was integrated at the same insertion site as the Rbp1 RNAi line (attP40).** n > 884 flies per group, cox proportional hazard mixed effects model p-value = 0.31 and log hazard ratio = -0.113 ± 0.110.
